# Variation of Transaminases, HCV-RNA Levels and Th1/Th2 Cytokine Production during the Post-Partum Period in Pregnant Women with Chronic Hepatitis C

**DOI:** 10.1371/journal.pone.0075613

**Published:** 2013-10-10

**Authors:** Ángeles Ruiz-Extremera, José Antonio Muñoz-Gámez, Ana Abril-Molina, María Angustias Salmerón-Ruiz, Paloma Muñoz-de-Rueda, Esther José Pavón-Castillero, Rosa Quiles-Pérez, Ángel Carazo, Ana Gila, Sergio Manuel Jimenez-Ruiz, Jorge Casado, Ana Belén Martín, Laura Sanjuán-Núñez, Esther Ocete-Hita, Julián López Viota, Josefa León, Javier Salmerón

**Affiliations:** 1 Paediatric Unit, San Cecilio University Hospital, Granada, Spain; 2 Centro de Investigación Biomédica en Red de Enfermedades Hepáticas y Digestivas, Ciberehd, Granada, Spain; 3 Paediatric Unit, Granada University, Granada, Spain; 4 Clinical Management Unit of Digestive Disease, San Cecilio University Hospital, Granada, Spain; 5 Paediatric Unit, Virgen de las Nieves Hospital, Granada, Spain; 6 Paediatric Unit, La Paz University Hospital, Madrid, Spain; 7 Medicine Department, Granada University, Granada, Spain; University of Cincinnati College of Medicine, United States of America

## Abstract

This study analyses the evolution of liver disease in women with chronic hepatitis C during the third trimester of pregnancy and the post-partum period, as a natural model of immune modulation and reconstitution. Of the 122 mothers recruited to this study, 89 were HCV-RNA+ve/HIV-ve and 33 were HCV-RNA-ve/HIV-ve/HCVantibody+ve and all were tested during the third trimester of pregnancy, at delivery and post-delivery. The HCV-RNA+ve mothers were categorized as either *Type-A* (66%), with an increase in ALT levels in the post-partum period (>40 U/L; P<0.001) or as *Type-B* (34%), with no variation in ALT values. The Type-A mothers also presented a significant decrease in serum HCV-RNA levels in the post-delivery period (P<0.001) and this event was concomitant with an increase in Th1 cytokine levels (INFγ, P = 0.04; IL12, P = 0.01 and IL2, P = 0.01). On the other hand, the Type-B mothers and the HCV-RNA-ve women presented no variations in either of these parameters. However, they did present higher Th1 cytokine levels in the partum period (INFγ and IL2, P<0.05) than both the Type-A and the HCV-RNA-ve women. Cytokine levels at the moment of delivery do not constitute a risk factor associated with HCV vertical transmission. It is concluded that differences in the ALT and HCV-RNA values observed in HCV-RNA+ve women in the postpartum period might be due to different ratios of Th1 cytokine production. In the Type-B women, the high partum levels of Th1 cytokines and the absence of post-partum variation in ALT and HCV-RNA levels may be related to permanent Th1 cytokine stimulation.

## Introduction

Infection with hepatitis C virus (HCV) is a worldwide health problem, affecting over 170 million persons, and is the most common cause of cirrhosis, hepatocellular carcinoma and liver transplantation [1], [2]. Its prevalence in pregnant women is similar to that found among the general population. In Spain, the incidence of HCV infection among pregnant women is estimated at 0.53–1.4% [3]–[5], which is in line with values reported for other industrialized countries [6]–[8]. Although HCV affects a significant number of pregnant women, few studies have actually examined the role of chronic HCV infection on pregnancy outcomes, and the results presented are contradictory. Some studies have reported obstetrical complications in HCV-infected pregnant women (premature rupture of membranes, preterm delivery, low new-born weight, cholestasis) [9]–[12], but others have concluded that, in the absence of cirrhosis and portal hypertension, most HCV-infected pregnant women do not experience obstetric complications [6], [13], [14]. During pregnancy, the treatment of HCV is contraindicated, and therefore there are no antiviral treatment recommendations for HCV-infected women during pregnancy, or guidelines for the prevention of vertical transmission, even though perinatal transmission is associated with a higher incidence of chronic liver disease [15]. Parity, hormone replacement therapy for postmenopausal women and the female sex have all been reported as protection factors against the progression of liver disease to cirrhosis, because the female hormones (oestrogen, progesterone) might play a role in delaying the onset of hepatic fibrosis [16]–[19]. Nonetheless, the natural history of chronic HCV infection during pregnancy and the puerperium has not been clearly established. In this period, the maternal immune system must develop a tolerance to paternal alloantigens in order to prevent maternal immune aggressions against the foetus and maintain active immunity against infectious agents to protect both the mother and foetus [20] For this reason, during pregnancy, the T helper 1 (Th1) associated cellular immune response is repressed and Th2-type immunity is stimulated in response to pathogens [21], [22]. These data suggest that the reduced hepatic damage detected in chronic HCV pregnant women (measured as low ALT levels) may be mediated by a modified Th1 immune response during pregnancy [8]; however, it should be noted that very few data have been reported and that many unanswered questions remain in this respect.

The vertical transmission of HCV (HCV-VT) is the major cause of paediatric HCV infection, and in industrialized countries it is the most common cause of chronic liver disease in children. Although the persistent transmission of HCV from infected mothers to their infants is reported in 4–8% of cases (chronically HCV-infected children), transient HCV perinatal infection also occurs, with an incidence of about 14–17% [23], [24].

Our clinical experience with pregnant women has shown that not all HCV-RNA+ve mothers have the same ALT pattern; some women appear to be “immune-tolerant” to HCV during pregnancy and the puerperium, presenting non-significant differences in viral load values together with persistently normal ALT levels. This finding led us to analyse the evolution of transaminases, HCV-RNA serum levels and Th1/Th2 cytokine production. Our study provides new insights into changes in ALT and HCV-RNA levels and a better understanding of the modulation of Th1/Th2 cytokine production in pregnant women with chronic hepatitis C.

## Materials and Methods

### Subjects

A prospective cohort study was conducted at San Cecilio University Hospital in Granada (Spain) from 1991 until 2009, where mothers were routinely tested for HCV during prenatal care (by the same doctors and with the same protocols). All patients included in this study were Caucasian race. The exclusion criteria were evidence of hepatitis B virus, HIV, alcoholism or autoimmune disease. The 122 mothers recruited to this study were classified into two groups: HCV-RNA+ve/HIV-ve (referred to as HCV-RNA+ve, n = 89) and HCV-RNA-ve/HIV-ve/HCVantibody+ve (control group, referred to as HCV-RNA-ve, n = 33). No obstetric complications were detected. The mothers were followed up in the Hepatology Unit of our Hospital and during the study period did not receive any immunomodulatory treatment (direct or indirect), such as steroids, IFN therapy, anti-inflammatory therapy, antibiotics or hormonal therapy. The mothers were tested for the study parameters during the third trimester of pregnancy (from week 28 of gestation to the end of the pregnancy), at delivery and during the post-partum period. Of the ALT determinations obtained for each post-partum group, the highest one in each case was used to categorize the women as Type-A or Type–B. The infants were examined by paediatricians and tested for HCV-RNA at birth and at 2, 4, 6, 8, 10, 12, 18 and 24 months, and thereafter at 3, 4, 5 and 6 years. IL28B (IFNL3) polymorphism (rs12979860) was determined in the infants and in their mothers. The HCV-RNA+ve women were then classified according to their ALT values in the post-partum period (3-6 months post-delivery) as either *Type-A*: with raised ALT levels in the postpartum period (ALT^Postpartum^>40 U/L) or as *Type-B*: with unchanged ALT values (ALT^Postpartum^≤40 U/L). The normal range of values for ALT was 10–40 U/L. To account for these differences, we studied the levels of HCV-RNA and of Th1 cytokines (INFγ, IL12 and IL2) and of Th2 cytokines (IL10 and IL4). The diagnosis of HCV-VT was based on detectable HCV-RNA in the peripheral blood by the polymerase chain reaction (PCR), defined as infants who presented HCV-RNA+ve in at least two subsequent blood samples. This criterion was established to minimize the risk of false positives. The persistent infection group of chronically HCV-infected children was defined as those children with persistent HCV-RNA+ve with HCV serum-conversion (detectable anti-HCV). Informed written consent was obtained in all cases (from each mother and by them on behalf of their children), and the study protocol conformed to the ethical guidelines of the 1975 Helsinki Declaration, as reflected in the *a priori* approval granted by the ethics committee of the San Cecilio University Hospital (Granada, Spain).

### ALT determination

The Clinical Biochemistry Laboratory at the San Cecilio University Hospital performed serum ALT measurements during the three months before delivery, at delivery and at 3–6 and 7–12 months post-partum.

### Virologic assays

HCV genotyping was determined by reverse hybridization (Inno-LIPA II HCV Innogenetics SA Ghent, Belgium) by the Clinical Biochemistry Laboratory of the San Cecilio University Hospital. Quantitative measurement of viral load (cut-off <15 IU/mL) was carried out using the HCV Ampliprep TaqMan, Roche Molecular System, at the same time as ALT determination.

### Cytokine quantification

Th1 cytokine response (INFγ, IL-12 and IL-2) and Th2 (IL-10, IL-4) were quantified at delivery and at 3–6 months post-partum in the serum of the mothers. These parameters were measured simultaneously using multiple immunoassay kits (Procarta® Cytokine Assay Kit, Quantitative Biology, Affymetrix®), following the manufacturer's instructions and using the Luminex® Technology. Quantitative data were obtained using the Luminex-200 system (Luminex Corporation, Austin, TX), and the data were analysed using Luminex 100 ™ IS v2.3 software. The sensitivity of the analytes tested was 0.12 pg/mL (High Sensitivity Human Cytokine Panel). To increase the sensitivity of the kit to this value (0.12 pg/mL), the manufacturer advised us to introduce a new standard with a lower concentration of the analyte (they recommended using 9 standards instead of 8). All the HCV-RNA+ve mothers included in the study presented detectable and quantifiable levels of these cytokines. Some of the HCV-RNA-ve mothers presented detectable not quantifiable levels of some cytokines. In these cases, the standard sensitivity of the kit was used. The numbers of the HCV-RNA-ve mothers with unquantifiable levels were: IL12: 6/33, IL2:5/33 and IL10: 2/33.

### IL28B (INFL3) genotyping

Rs12979860 genotyping was performed by means of a Taqman 5′ allelic discrimination assay (Custom Assay Service, Applied Biosystems, Foster City, CA, USA) as described previously [24], [25].

### Statistical Analysis

Data management and analysis were performed using SPSS 15.0 for Windows. The criterion for statistical significance was P-value ≤ 0.05. Qualitative variables are expressed as absolute values with percentages, and quantitative variables are expressed as mean values ± SEM (standard error of the mean). Comparisons between groups for categorical variables were made by the χ2 and Fisher's exact test. For differences in the quantitative variables, the paired/unpaired Student's t test for normally distributed variables and the Mann-Whitney Test for variables with a non-normal distribution were applied. The Kolmogorov-Smirnov test was used to analyse the distribution of the quantitative variables. Pearson's correlation was employed to analyse the degree of linear relationship between two variables.

## Results

### Study cohort

The characteristics of the mothers included in this study are described in Table S1. There were no significant differences in median age, gestational age (period of time between conception and birth), type of birth, new-born weight, etc. However, of the 89 HCV-RNA+ve mothers, 21 (23%) had IL28B CC polymorphism whereas among the 33 HCV-RNA-ve women (control group), 20 (61%) had IL28B CC polymorphism (P>0.01). Accordingly, the mothers with non-CC IL28B polymorphism had a greater probability of being HCV-RNA+ve than did those with CC polymorphism (OR = 3.01; 95%CI: 1.3–5.7; P = 0.02). 74% of the HCV-RNA+ve mothers had been infected by parenteral route. The mean value of the viral load in the HCV-RNA+ve mothers was 2,709,588 IU/mL and 75% of these mothers had viral genotype 1. Moreover, 54% of the HCV-RNA+ve women had high viral load (>600,000 UI/mL) at delivery.

### ALT and cytokine evolution in HCV-RNA+ve *vs*. HCV-RNA-ve mothers

**Figure 1:**
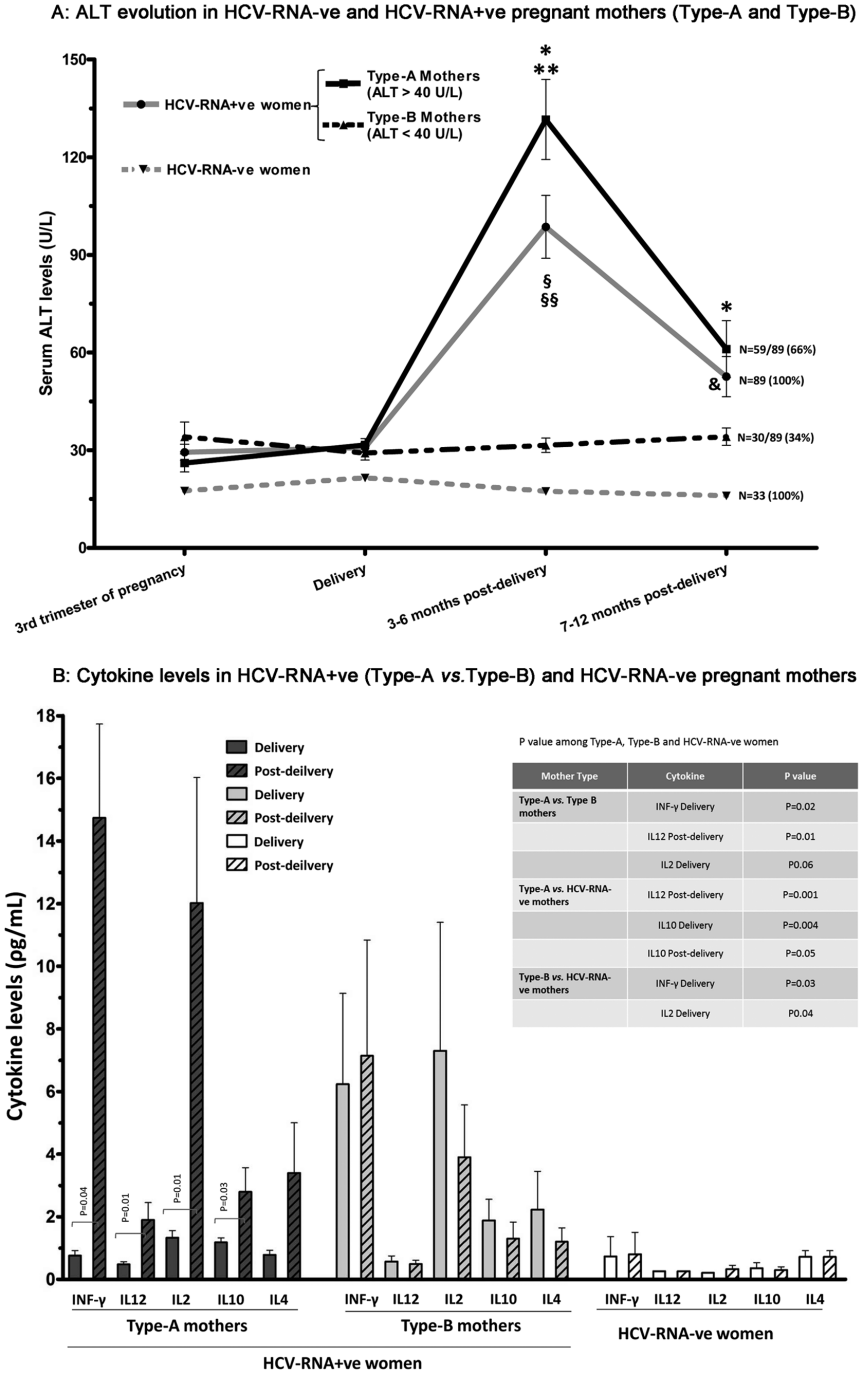
Evolution of ALT (A) and cytokine serum levels (B) in HCV-RNA-ve and HCV-RNA+ve pregnant women (categorized into Type-A and Type-B mothers). Statistical analysis was performed using the paired/unpaired Student's t test for normally distributed quantitative variables and the Mann-Whitney Test for quantitative variables with a non-normal distribution. The Kolmogorov-Smirnov test was used to analyse the distribution of quantitative variables. ^§^P<0.01 comparing HCV-RNA+ve and HCV-RNA-ve mothers. ^§§^P<0.01 comparing HCV-RNA+ve mothers, 3–6 months post-partum, at delivery and at 7–12 months post-delivery.*P<0.01 comparing Type-A and Type-B HCV-RNA+ve mothers. **P<0.001 comparing Type-A mothers at 3-6 months post-partum, at delivery and at 7–12 months post-partum.

The evolution of ALT in the HCV-RNA+ve and in the HCV-RNA-ve women is shown in Figure 1A. Interestingly, non-significant differences in ALT values were observed between these groups during the third trimester of pregnancy (from week 28 of gestation to the end of the pregnancy), and at delivery. However, after delivery (3–6 months post-partum), the HCV-RNA+ve mothers showed higher ALT levels than did the HCV-RNA-ve mothers (98.32±9.34 U/L *vs*. 17.42±1.77 U/L; P<0.001). Moreover, the HCV-RNA+ve mothers presented a significant increase in Th1 cytokines at 3–6 months post-partum (ΔINFγ = INFγ^post-partum^–INFγ^partum^ = 9.1±2.5ρg/mL, P = 0.03; ΔIL12 = IL12^post-partum^–IL12^partum^ = 0.85±0.30ρg/mL, P = 0.01 and ΔIL2 = IL2^post-partum^–IL2^partum^ = 5.4±2.89ρg/mL, P = 0.07) whereas in the HCV-RNA-ve mothers the cytokine values in the peripheral blood remained unchanged (however this does not mean that such differences do not exist in the placenta, decidua and umbilical cord). Furthermore, the HCV-RNA+ve mothers had higher cytokine levels than the HCV-RNA-ve mothers, both at delivery and 3–6 months post-partum. On the other hand, in the HCV-RNA+ve mothers there was no significant association between cytokine levels and IL28B polymorphism (CC *vs* non-CC) or between cytokine levels and viral genotype [geno. 1 (1a *vs* 1b) and *vs* geno. non-1 (3 and 4)].

### Variations in ALT levels in HCV-RNA+ve mothers: Type-A and Type-B women

Not all the HCV-RNA+ve mothers presented the same patterns with respect to the parameters analysed. Therefore, and as described in the Experimental Procedures section, these women were categorized as either *Type-A* (59/89; 66%): raised serum ALT levels in the post-partum period (ALT^3–6 months post-partum^>40 U/L and an average increase in ALT level calculated as ΔALT = ALT^3–6 months post-partum^–ALT^partum^ = 100±7 U/L; P<0.001), or *Type-B* (30/89; 34%): unchanged ALT values (ALT^postpartum^≤40 U/L) (Figure 1A). The Type-A women, at 7–12 months post-partum, presented a significant decrease in ALT values (ΔALT = ALT^3–6 months post-partum^–ALT^7–12 months post-partum^ = 70.52±10.49 U/L; P<0.01). Interestingly, the Type-B mothers had a similar ALT pattern to that presented by the HCV-RNA-ve women (Figure 1A).

### Characteristics of the Type-A and Type-B mothers

The characteristics of the Type-A and B mothers are described in Table 1. Some clinical characteristics, such as median age, viral genotype, IL28B polymorphism, type of birth, breast-fed infants, breast-feeding days, epidemiology, gestational age, etc., were similar, but important features that might account for the categorization in each case were also found; thus, 38/59 (64%) of the Type-A mothers had high HCV viral load at delivery (>600,000 IU/mL) in contrast to 10/30 (33%) of the Type-B women (P = 0.01). Furthermore, the Type-A mothers presented a significantly lower viral load after delivery than the Type-B mothers (ΔVL = VL ^3–6 months^ ^post-partum^–VL^partum^ = -2.438.867±643.942 IU/mL *vs*. 11.288±664.352 IU/mL respectively; P = 0.031). Another interesting aspect is the relation between cytokine levels and type of mother (Table 1 and Figure 1B). Thus, the Type-A mothers at 3–6 months post-partum presented higher values for INFγ, IL12 and IL2 (Th1 cytokine), as well as for IL10 (Th2 and anti-inflammatory cytokine). The largest such increases took place in the Th1 cytokines INFγ and IL2. It is known that before and during delivery, the levels of oestradiol and progesterone may vary. In order to determine the possible effect on cytokine production, we studied the Th1/Th2 cytokines in the control group (HCV-RNA-ve mothers). No partum effect was detected (Figure 1B), and therefore whatever change took place in the female hormones during the intra-partum period had no effect on cytokine production. On the other hand, the Type-B mothers did not show this cytokine behaviour pattern and no statistically significant data were obtained. These Type-B mothers had higher INFγ and IL2 levels at the moment of delivery than did the HCV-RNA-ve and Type-A mothers (Th1 cytokines, Figure 1B, P>0.05).

**Table 1:** Characteristics of the Type-A and Type-B mothers.

	Variable	Type-A mothers n = 59 (66%)	Type-B mothers n = 30 (34%)	*P-Value*
**Mother characteristic**	Age^1^	29±0.7	30±0.9	ns
	Gestational age^1^ (weeks)	38.84±0.3	38.80±0.4	ns
	IL28B genotype^2^ (CC)	15 (25)	6 (20)	ns
	Epidemiology^2^ (Parenteral)	42 (71)	24 (80)	ns
**Viral characteristic**	Viral Genotype^2^ (Geno. 1)	45 (76)	22 (73)	ns
	Viral Genotype^2^ (Geno. 3)	12 (20)	7 (23)	ns
	Viral Genotype^2^ (Geno. 4)	2 (0.3)	1 (0.3)	ns
	Delivery viral load^2^ (>600,000 IU/mL)	38 (64)	10 (33)	0,01
	VL^delivery^ –VL^post-childbirth (3–6 months)1^	−2.438.867±643.942	11.288±664.352	0.031
	HCV-RNA spontaneous clearance^2^	2 (3)	2 (6)	ns
**Child characteristic**	Weight (g)^1^	3216±68	3025±105	ns
	Vertically transmitted^2^ (yes)	12 (20)	3 (10)	ns
	Type of birth^2^ (Caesarean)	11 (19)	8 (27)	ns
	Breast-fed^2^ (yes)	42 (71)	23 (77)	ns
	Breast-feeding days^1^	50±8	60±15	ns
**Cytokine values** 3	INFγ	14.0±6.6	0.91±1.45	0.06
	IL12	1.41±0.51	−0.09±0.16	0.008
	IL2	10.69±3.98	−3.38±3.00	0.016
	IL10	1.62±0.72	−0.59±0.57	0.037
	IL4	2.62±1.60	−1.03±0.96	ns

1 Mean ± the standard error of the mean (SEM) ns  =  non-significant

2 Values are absolute with percentages in parentheses VL = Viral Load

3 3–6 months post-partum-delivery; ρg/mL

Bivariate analysis: P-value by chi-squared test for qualitative variables and Student's t test for normally distributed quantitative variables and the Mann-Whitney Test for quantitative variables with a non-normal distribution.

### ALT and HCV viral load evolution in both types of HCV-RNA+ve pregnant women

**Figure 2:**
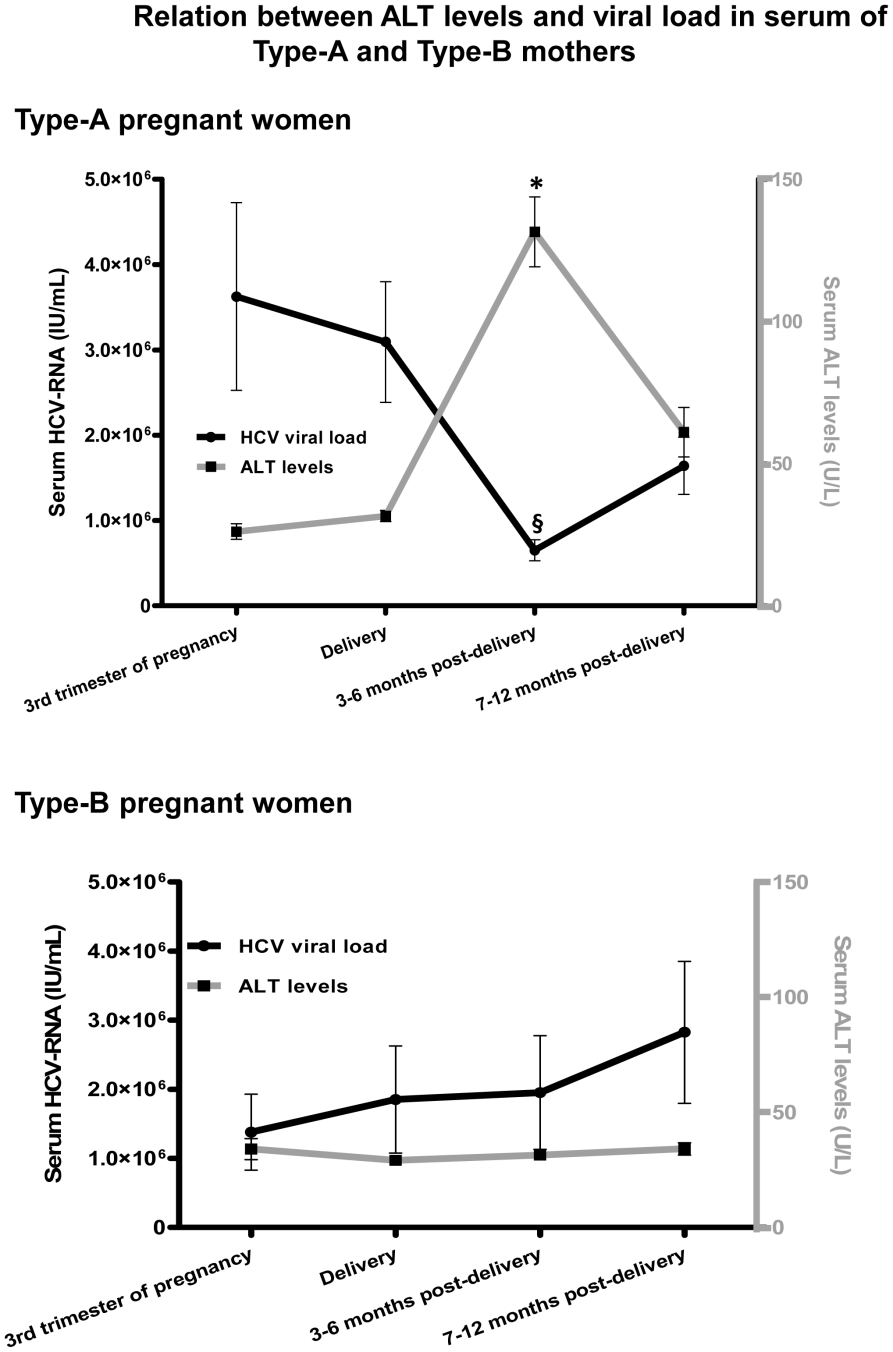
Relation between ALT levels and HCV viral load in the serum of Type-A and Type-B mothers. Statistical analysis was performed using the paired/unpaired Student's t test for normally distributed quantitative variables and the Mann-Whitney Test for quantitative variables with a non-normal distribution. The Kolmogorov-Smirnov test was used to analyse the distribution of quantitative variables. *, ^§^ P<0.001, comparing the third trimester of pregnancy, delivery and 7–12 months post-partum.

Analysis of ALT and HCV viral load revealed a temporal correlation among the Type-A mothers (Figure 2); ALT levels had increased and the viral load had decreased at 3–6 months post-partum (Figure S1; P<0.0001; Pearson's R = −0.740). However, the Type-B women did not present this correlation (Figure S1; P = 0.234; Pearson's R = 0.234) Furthermore, analysis of the HCV viral load in the Type-A women (Figure S2) showed that both in the mothers with high HCV-RNA viral load (>600,000 IU/mL) and in those with low HCV-RNA levels at delivery (≤600,000 IU/mL), there was a significant decrease in the HCV viral load. This post-partum decrease was independent of the HCV-RNA values at delivery. However, the Type-A women, whether with high or low viral load at delivery, presented raised INFγ, IL12 and IL2 levels post-partum (compared with the partum values, Th1 cytokines, Figure S3) and higher levels of IL10 (Th2 and anti-inflammatory cytokines). These data suggest that the cytokine behaviour pattern has a stronger influence on ALT evolution at delivery and 3–6 months post-partum than does the viral load at the same times. On the other hand, the Type-B women classified according to viral load at delivery did not present this behaviour pattern with respect to the evolution of cytokine levels in the peripheral blood.

### HCV vertical transmission

**Figure 3:**
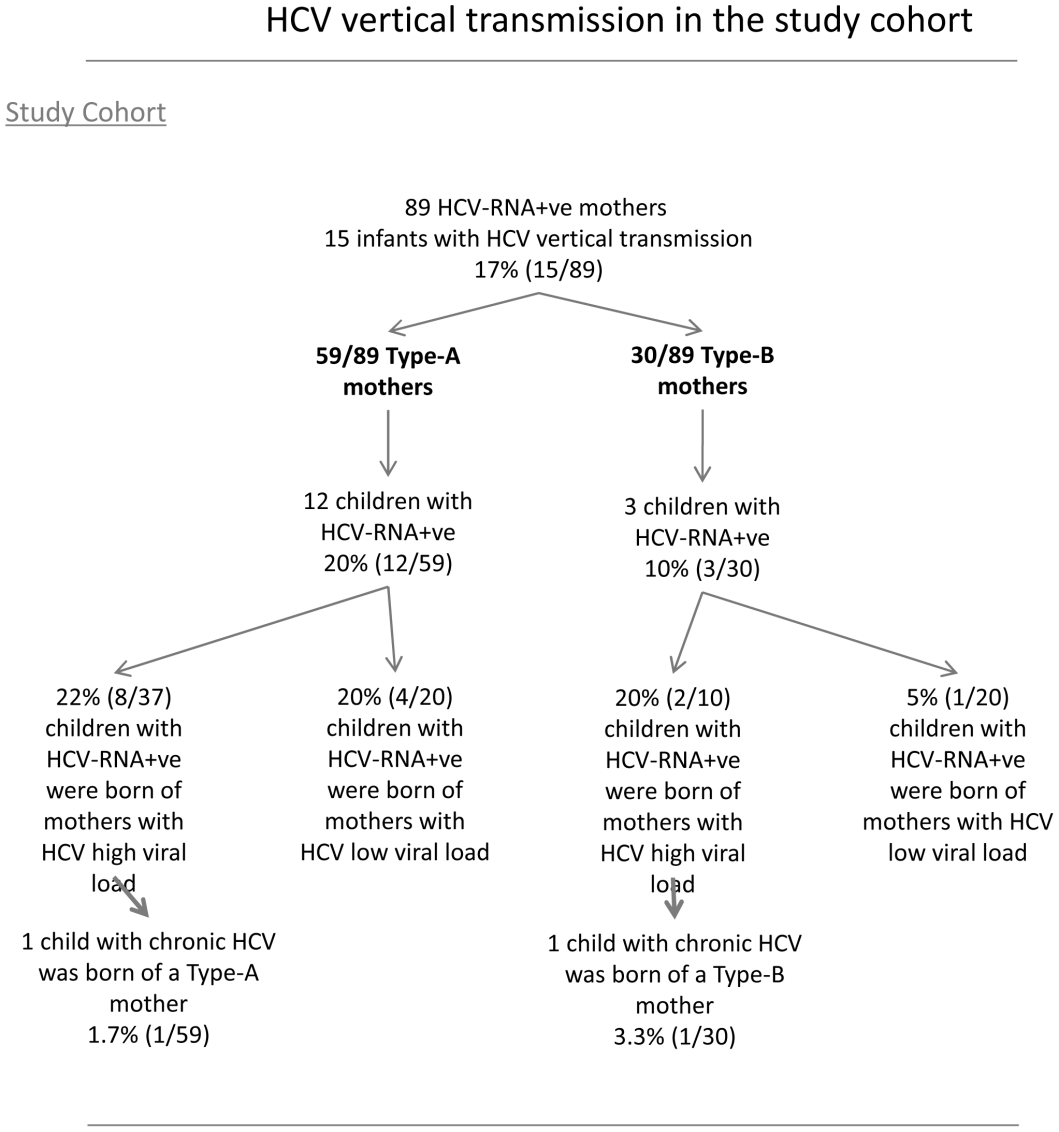
Description of HCV vertical transmission in the study cohort, showing the data for the 89 HCV-RNA+ve women (categorized into Type-A and Type-B mothers).

The analysis of cytokine levels at delivery did not reveal any statistically significant association with HCV vertical transmission (HCV-VT). The categorization of mothers into Types-A and B showed that of the 15 infants suffering vertically transmitted HCV, 12 were born of Type-A mothers and only 3 of Type-B mothers; nevertheless, the difference was not statistically significant (Table 1 and Figure 3). Interestingly, when we categorized the Type-A and Type-B mothers in accordance with their viral load values (high or low), the Type-B mothers with low viral load had the lowest probability of HCV vertical transmission (5% in comparison to 20% in the rest of the HCV-RNA+ve women, Figure 3). In relation to chronically HCV-infected children, only two infants were HCV-RNA+ve at the end of the study (2 of the 89 children were born of HCV-RNA+ve mothers, 2.2%) and no statistically significant data were obtained. It is noteworthy that the IL28B genotype observed in the two chronically HCV-infected infants was non-CC.

### HCV-RNA spontaneous clearance in pregnant mothers

In our study cohort, several spontaneous HCV clearances [4 of the 89 women (4.4%)] were observed, at 3–6 months after delivery (Table S2), but the factors involved are unknown. Three of these four women had low viral load and IL28B genotype non-TT (two women were CC and one was CT). In this respect, no differences were detected between Type-A and Type-B mothers. One mother suffered a relapse some months after the spontaneous clearance.

## Discussion

The aim of this study was to determine ALT evolution in women with chronic hepatitis C during the third trimester of gestation, at delivery and in the post-partum period, as a natural model of immune modulation and reconstitution; accordingly, we analysed the relations between serum ALT levels, the HCV-RNA viral load and Th1/Th2 cytokine production.

To date, only a few studies, with small sample populations, have considered the evolution of HCV during both pregnancy and the post-delivery period. Therefore, much remains unknown in this respect. Our study addresses this issue using a representative cohort of HCV-RNA+ve and HCV-RNA-ve pregnant women. The cohort was monitored in regular follow-ups, from the third trimester of gestation until one year after delivery. In the HCV-RNA+ve mothers, during the third trimester of gestation and at delivery, non-significant differences in ALT values were observed. Moreover, the ALT values presented were analogous with those for the HCV-RNA-ve mothers (ALT≤40 U/L). These data are in accordance with the findings of Gervais et al. [26] and Paternoster et al. [27], who reported decreased ALT levels in serum during the second and third trimesters of pregnancy until normal ALT values (ALT≤40 U/L), together with a corresponding increase in HCV-RNA levels. We show that not all HCV-RNA+ve mothers present the same behaviour patterns in the puerperium for the parameters analysed (essentially, the ALT levels). For this reason, the women were classified into two groups: Type-A (66%) with increased ALT levels in the post-partum period, and Type-B (34%), with no variation in these ALT values. To account for these differences, we evaluated HCV-RNA and Th1/Th2 cytokine levels in the peripheral blood. The Type-A mothers presented a significant decrease in serum HCV-RNA levels in the post-partum period, and this event was concomitant with an increase in Th1 cytokine levels. Moreover, in the HCV-RNA+ve mothers as well as in each of the two subgroups (Types A and B), there was no significant association between the HCV viral genotype vs. the ALT and vs. cytokine levels. On the other hand, Lin et al. [28], in a study of ten pregnant women, and Hattori [29], who studied 22 pregnant women, found that HCV-RNA levels tend to decrease in the post-partum period. However, neither of these studies provided a definitive immune interpretation of these events. It has been widely reported that in the general population (but not among pregnant women with chronic HCV), the Th1 response is related to viral clearance and treatment response, while that of Th2 is related to HCV chronicity [30]. Thus, in the Th1 immune response against HCV, the IL12 released from dendritic cells promotes a cell-mediated response, eliciting a secretion of INF-gamma by NK and T cells. This event favours neutrophil and macrophage recruitment and provokes an inflammatory response. Therefore, the post-delivery stimulation of Th1 immune-response observed in the Type-A women may explain the decrease in HCV-RNA viral load and the increase in the ALT levels as the result of a hepatic injury. In our opinion, this period may be an optimal time to initiate antiviral treatment in Type-A women who failed to respond to previous treatment, because this Th1 re-stimulation might augment their natural defence mechanisms. IL10 is a potent anti-inflammatory Th2 cytokine, mainly produced by monocytes, macrophages and T cells, and it is capable of inhibiting the synthesis of pro-inflammatory cytokines such as IFN-γ and IL-2. Moreover, it has been reported that increased levels of IL-4 can promote the conversion of macrophages into an M2 phenotype (also termed repair macrophages or anti-inflammatory M2 macrophages) and inhibit the activation of M1 macrophages (inflammatory M1 macrophages). Increased levels of repair macrophages (M2) are associated with the secretion of IL-10 and TGF-β, which reduce pathological inflammation [31]. Consequently, we consider that the slight increase in IL10 (P = 0.03) and in IL4 (non-significant, P>0.05), might be an attempt to reduce the intense inflammatory response triggered by Th1 cytokines. These data suggest that treatment with Th1 cytokines might have beneficial results in HCV therapy. Currently, only INF-alpha is used in the treatment of chronic viral hepatitis because the effectiveness of the exogenous administration of IL12, IL2 and IL10 is unclear [30]. On the other hand, in the HCV-RNA-ve mothers the cytokine values in the peripheral blood remained unchanged because the mothers' immunity was not stimulated in response to HCV.

In the present study, the Type-B mothers were studied in detail. These women presented no variations in any of the parameters analysed and appeared to react as “immune-tolerant” to HCV (women with non-significant differences in their viral load values, together with persistently normal ALT levels). However, when we analysed the Th1/Th2 cytokine production, our initial opinion was modified, because surprisingly, the Type-B women were found to have a stimulated immune system with high levels of INF-γ and IL-2 (Th1 cytokines) both before and after delivery. Interestingly, several studies of the general population of chronically HCV infected persons have reported high baseline levels of Th1 cytokines (IL-2, INF-γ and TNF-α) and the expression of interferon-stimulated genes, together with an absence of post-treatment variation in the serum of patients without sustained viral response (non-SVR) [32]–[35]. We consider that in the Type-B mothers, as in non-SVR patients, the activation of the endogenous Th1 system is unable to reduce the levels of the HCV infection and that this may be associated with an ineffective or exhausted antiviral response. However, the biological mechanism responsible for the high baseline expression remains unclear. Recent studies have suggested that baseline expression is associated with IL28B polymorphism [36], [37], although others have concurred with us in finding no such association [38], [39].

In relation to the vertical transmission of HCV, no relation was observed with Th1/Th2 cytokine levels in the peripheral blood. These data are in accordance with the findings of Paternoster et al., who reported that the levels of endogenous interferon-alpha during pregnancy were not related to the vertical transmission of HCV [40]. However, it was recently reported that the placenta constitutes an active innate immunological organ that provides antiviral protection against HCV transmission [41]. These authors described several locally acting mechanisms in HCV-exposed placenta, such as the increased production of cytokines by NK cells (IFN-β, SLPI, etc.) and the cytotoxicity triggered by NK T cells [41], which protect the foetus against HCV. These data might complement those observed at the systemic level (modulation in the Th1 immune response). Further studies are needed to better understand the complexities of the immune system during the pregnancy and its relation with the vertical transmission of HCV. We identified a group of pregnant women with the lowest HCV-VT rate. These mothers were characterized by low viral load and by the absence of any increase in ALT levels after delivery. These findings are in accordance with those of previous studies, which have identified the main risk factors for perinatal mother-to-child transmission of HCV as the high concentration of HCV-RNA in maternal blood and HIV co-infection [25]. Two studies have shown that high ALT levels in the serum during pregnancy and at delivery are associated with higher rates of HCV-VT [42], [43]. Due to the low number of chronically HCV-infected children in our study cohort, no statistically significant data in this respect were obtained; nevertheless, an interesting point was that the IL28B genotype observed in the two chronically HCV-infected infants was non-CC, a finding that is in accordance with our own previously published data [24].

All retrospective analyses have inherent limitations, but we have tried to minimize these effects. The standard method of HCV determination was modified during the patient inclusion period, but this factor was controlled by using the same PCR technique (HCV Ampliprep TaqMan, Roche Molecular System) on all the patients studied, using a stored blood sample. The standard care procedure for HIV and HCV patients was also modified during the patient inclusion period; however, in this study the risk factors for the HIV-ve mothers (Study Cohort) were identified. According to standard protocols for HCV-RNA+ve pregnant women, no HCV treatment should be applied during pregnancy, and thus the changes in standard care for HCV patients do not affect our study.

In conclusion, the different values for ALT (in the post-partum period) and HCV-RNA (at delivery and in the post-partum period) observed in the HCV-RNA+ve women might be due to different ratios of Th1 cytokine production. Thus, the Type-A mothers presented a significant decrease in serum HCV-RNA levels in the post-partum period and this event was concomitant with an increase in Th1 cytokine levels. On the other hand, in the Type B women, the high levels of Th1 cytokines observed at delivery, together with the unchanged levels of ALT and HCV-RNA post-partum, may be related to the existence of permanent Th1 cytokine stimulation. Finally, the cytokine values in the HCV-RNA-ve mothers did not change, because their immunity was not stimulated against HCV. The findings of this study could enhance our understanding of the natural history of chronic hepatitis C infection during pregnancy and the puerperium, and help identify mothers at low risk of vertical HCV infection, which would be useful for the development of prevention strategies.

## Supporting Information

Figure S1 Measure of the association between ΔALT and ΔViral load. The HCV-RNA+ve mothers exhibited a significant correlation between ALT levels and viral load, while the Type-B women did not present this association. *ΔViralLoad = VL^3–6 months post-partum^–VL^partum^*, ΔALT: ALT^3-6 months post-partum^–ALT^partum^. Statistical analysis was performed using Pearson's r to measure the correlation (linear dependence) between the two variables.
(TIF)

Figure S2 HCV-RNA+ve women classified according to their viral load. Mothers with high viral load: >600,000 IU/mL; mothers with low viral load: ≤600,000 IU/mL. Statistical analysis was performed using the paired/unpaired Student's t test for normally distributed quantitative variables and the Mann-Whitney Test for quantitative variables with a non-normal distribution. The Kolmogorov-Smirnov test was used to analyse the distribution of quantitative variables.
(TIF)

Figure S3 The evolution of the cytokine serum levels in HCV-RNA+ve pregnant women (Type-A *vs*. Type-B) categorized into high viral load and low viral load according to the serum HCV-RNA levels in the intra-partum period. Mothers with high viral load: >600,000 IU/mL; mothers with low viral load: ≤600,000 IU/mL. Statistical analysis was performed using the paired/unpaired Student's t test for normally distributed quantitative variables and the Mann-Whitney Test for quantitative variables with a non-normal distribution.
(TIF)

Table S1 Characteristics of HCV-RNA-ve and HCV-RNA+ve mothers.
(TIF)

Table S2 Characteristics of the mothers with HCV-RNA spontaneous clearance during pregnancy and/or puerperium.
(TIF)

## References

[1] Pol S, Vallet-Pichard A, Corouge M, Mallet VO (2012) Hepatitis C: epidemiology, diagnosis, natural history and therapy. Contrib Nephrol 176: 1–9. 22310776 10.1159/000332374

[2] Seeff LB (2002) Natural history of chronic hepatitis C. Hepatology. 36: S35–46. 10.1053/jhep.2002.36806 12407575

[3] Salmeron J, Gimenez F, Torres C, Ros R, Palacios A, et al (1998) Epidemiology and prevalence of seropositivity for hepatitis C virus in pregnant women in Granada. Rev Esp Enferm Dig 90: 841–850. 9973846

[4] Ruiz-Extremera A, Salmeron J, Torres C, De Rueda PM, Gimenez F, et al (2000) Follow-up of transmission of hepatitis C to babies of human immunodeficiency virus-negative women: the role of breast-feeding in transmission. Pediatr Infect Dis J 19: 511–516. 10877164 10.1097/00006454-200006000-00004

[5] Ruiz-Extremera A, Lopez-Garrido MA, Barranco E, Quintero MD, Ocete-Hita E, et al (2005) Activity of hepatic enzymes from week sixteen of pregnancy. Am J Obstet Gynecol 193: 2010–2016. 16325605 10.1016/j.ajog.2005.04.045

[6] Conte D, Fraquelli M, Prati D, Colucci A, Minola E (2000) Prevalence and clinical course of chronic hepatitis C virus (HCV) infection and rate of HCV vertical transmission in a cohort of 15,250 pregnant women. Hepatology 31: 751–755. 10706568 10.1002/hep.510310328

[7] Agha S, Sherif LS, Allam MA, Fawzy M (1998) Transplacental transmission of hepatitis C virus in HIV-negative mothers. Res Virol 149: 229–234. 9783338 10.1016/s0923-2516(98)80004-6

[8] Arshad M, El-Kamary SS, Jhaveri R (2011) Hepatitis C virus infection during pregnancy and the newborn period—are they opportunities for treatment? J Viral Hepat 18: 229–236. 21392169 10.1111/j.1365-2893.2010.01413.x

[9] Pergam SA, Wang CC, Gardella CM, Sandison TG, Phipps WT, et al. (2008) Pregnancy complications associated with hepatitis C: data from a 2003–2005 Washington state birth cohort. Am J Obstet Gynecol 199: : 38 e31–39. 10.1016/j.ajog.2008.03.052 PMC2517631 18486089

[10] Locatelli A, Roncaglia N, Arreghini A, Bellini P, Vergani P, et al (1999) Hepatitis C virus infection is associated with a higher incidence of cholestasis of pregnancy. Br J Obstet Gynaecol 106: 498–500. 10430202 10.1111/j.1471-0528.1999.tb08305.x

[11] Paternoster DM, Fabris F, Palu G, Santarossa C, Bracciante R, et al (2002) Intra-hepatic cholestasis of pregnancy in hepatitis C virus infection. Acta Obstet Gynecol Scand 81: 99–103. 11942897

[12] Safir A, Levy A, Sikuler E, Sheiner E (2010) Maternal hepatitis B virus or hepatitis C virus carrier status as an independent risk factor for adverse perinatal outcome. Liver Int 30: 765–770. 20214739 10.1111/j.1478-3231.2010.02218.x

[13] Floreani A, Paternoster D, Zappala F, Cusinato R, Bombi G, et al (1996) Hepatitis C virus infection in pregnancy. Br J Obstet Gynaecol 103: 325–329. 8605128 10.1111/j.1471-0528.1996.tb09736.x

[14] Jabeen T, Cannon B, Hogan J, Crowley M, Devereux C, et al (2000) Pregnancy and pregnancy outcome in hepatitis C type 1b. QJM 93: 597–601. 10984554 10.1093/qjmed/93.9.597

[15] Valladares G, Chacaltana A, Sjogren MH (2010) The management of HCV-infected pregnant women. Ann Hepatol 9 Suppl92–97. 20714003

[16] Lai JC, Verna EC, Brown RS Jr, O'Leary JG, Trotter JF, et al (2011) Hepatitis C virus-infected women have a higher risk of advanced fibrosis and graft loss after liver transplantation than men. Hepatology 54: 418–424. 21538434 10.1002/hep.24390 PMC3144983

[17] Hayashida K, Shoji I, Deng L, Jiang DP, Ide YH, et al (2010) 17beta-estradiol inhibits the production of infectious particles of hepatitis C virus. Microbiol Immunol 54: 684–690. 21044142 10.1111/j.1348-0421.2010.00268.x

[18] Di Martino V, Lebray P, Myers RP, Pannier E, Paradis V, et al (2004) Progression of liver fibrosis in women infected with hepatitis C: long-term benefit of estrogen exposure. Hepatology 40: 1426–1433. 15565616 10.1002/hep.20463

[19] Codes L, Asselah T, Cazals-Hatem D, Tubach F, Vidaud D, et al (2007) Liver fibrosis in women with chronic hepatitis C: evidence for the negative role of the menopause and steatosis and the potential benefit of hormone replacement therapy. Gut 56: 390–395. 17005762 10.1136/gut.2006.101931 PMC1856786

[20] Le Campion A, Larouche A, Fauteux-Daniel S, Soudeyns H (2012) Pathogenesis of hepatitis C during pregnancy and childhood. Viruses 4: 3531–3550. 23223189 10.3390/v4123531 PMC3528278

[21] Saito S (2000) Cytokine network at the feto-maternal interface. J Reprod Immunol 47: 87–103. 10924744 10.1016/s0165-0378(00)00060-7

[22] Sykes L, MacIntyre DA, Yap XJ, Ponnampalam S, Teoh TG, et al (2012) Changes in the Th1:Th2 cytokine bias in pregnancy and the effects of the anti-inflammatory cyclopentenone prostaglandin 15-deoxy-Delta(12,14)-prostaglandin J2. Mediators Inflamm 2012: 416739. 22690041 10.1155/2012/416739 PMC3368617

[23] Shebl FM, El-Kamary SS, Saleh DA, Abdel-Hamid M, Mikhail N, et al (2009) Prospective cohort study of mother-to-infant infection and clearance of hepatitis C in rural Egyptian villages. J Med Virol 81: 1024–1031. 19382251 10.1002/jmv.21480 PMC3235472

[24] Ruiz-Extremera A, Munoz-Gamez JA, Salmeron-Ruiz MA, de Rueda PM, Quiles-Perez R, et al (2011) Genetic variation in interleukin 28B with respect to vertical transmission of hepatitis C virus and spontaneous clearance in HCV-infected children. Hepatology 53: 1830–1838. 21413051 10.1002/hep.24298

[25] de Rueda PM, Lopez-Nevot MA, Saenz-Lopez P, Casado J, Martin-Casares A, et al (2011) Importance of host genetic factors HLA and IL28B as predictors of response to pegylated interferon and ribavirin. Am J Gastroenterol 106: 1246–1254. 21670772 10.1038/ajg.2011.82

[26] Gervais A, Bacq Y, Bernuau J, Martinot M, Auperin A, et al (2000) Decrease in serum ALT and increase in serum HCV RNA during pregnancy in women with chronic hepatitis C. J Hepatol 32: 293–299. 10707870 10.1016/s0168-8278(00)80075-6

[27] Paternoster DM, Santarossa C, Grella P, Palu G, Baldo V, et al (2001) Viral load in HCV RNA-positive pregnant women. Am J Gastroenterol 96: 2751–2754. 11569706 10.1111/j.1572-0241.2001.04135.x

[28] Lin HH, Kao JH (2000) Hepatitis C virus load during pregnancy and puerperium. BJOG 107: 1503–1506. 11192107 10.1111/j.1471-0528.2000.tb11675.x

[29] Hattori Y, Orito E, Ohno T, Sugauchi F, Suzuki S, et al (2003) Loss of hepatitis C virus RNA after parturition in female patients with chronic HCV infection. J Med Virol 71: 205–211. 12938194 10.1002/jmv.10471

[30] Fallahi P, Ferri C, Ferrari SM, Corrado A, Sansonno D, et al (2012) Cytokines and HCV-related disorders. Clin Dev Immunol 2012: 468107. 22611419 10.1155/2012/468107 PMC3352261

[31] Mercalli A, Calavita I, Dugnani E, Citro A, Cantarelli E, et al.. (2013) Rapamycin unbalances the polarization of human macrophages to M1. Immunology. 10.1111/imm.12126 PMC3784164 23710834

[32] Wan L, Kung YJ, Lin YJ, Liao CC, Sheu JJ, et al (2009) Th1 and Th2 cytokines are elevated in HCV-infected SVR(-) patients treated with interferon-alpha. Biochem Biophys Res Commun 379: 855–860. 19118522 10.1016/j.bbrc.2008.12.114

[33] Sarasin-Filipowicz M, Oakeley EJ, Duong FH, Christen V, Terracciano L, et al (2008) Interferon signaling and treatment outcome in chronic hepatitis C. Proc Natl Acad Sci U S A 105: 7034–7039. 18467494 10.1073/pnas.0707882105 PMC2383932

[34] Kottilil S, Yan MY, Reitano KN, Zhang X, Lempicki R, et al (2009) Human immunodeficiency virus and hepatitis C infections induce distinct immunologic imprints in peripheral mononuclear cells. Hepatology 50: 34–45. 19551908 10.1002/hep.23055 PMC2736098

[35] Lempicki RA, Polis MA, Yang J, McLaughlin M, Koratich C, et al (2006) Gene expression profiles in hepatitis C virus (HCV) and HIV coinfection: class prediction analyses before treatment predict the outcome of anti-HCV therapy among HIV-coinfected persons. J Infect Dis 193: 1172–1177. 16544259 10.1086/501365

[36] Naggie S, Osinusi A, Katsounas A, Lempicki R, Herrmann E, et al (2012) Dysregulation of innate immunity in hepatitis C virus genotype 1 IL28B-unfavorable genotype patients: Impaired viral kinetics and therapeutic response. Hepatology 56: 444–454. 22331604 10.1002/hep.25647 PMC3361636

[37] Urban TJ, Thompson AJ, Bradrick SS, Fellay J, Schuppan D, et al (2010) IL28B genotype is associated with differential expression of intrahepatic interferon-stimulated genes in patients with chronic hepatitis C. Hepatology 52: 1888–1896. 20931559 10.1002/hep.23912 PMC3653303

[38] Dill MT, Duong FH, Vogt JE, Bibert S, Bochud PY, et al (2011) Interferon-induced gene expression is a stronger predictor of treatment response than IL28B genotype in patients with hepatitis C. Gastroenterology 140: 1021–1031. 21111740 10.1053/j.gastro.2010.11.039

[39] Asahina Y, Tsuchiya K, Muraoka M, Tanaka K, Suzuki Y, et al (2012) Association of gene expression involving innate immunity and genetic variation in interleukin 28B with antiviral response. Hepatology 55: 20–29. 21898478 10.1002/hep.24623

[40] Paternoster DM, Belligoli A, Ngaradoumbe NK, Visentin S, Franco R, et al (2008) Endogenous interferon-alpha level is increased in hepatitis C virus (HCV)-positive pregnant women. J Clin Gastroenterol 42: 204–207. 18209593 10.1097/01.mcg.0000247991.81591.2e

[41] Hurtado CW, Golden-Mason L, Brocato M, Krull M, Narkewicz MR, et al (2010) Innate immune function in placenta and cord blood of hepatitis C—seropositive mother-infant dyads. PLoS One 5: e12232. 20814429 10.1371/journal.pone.0012232 PMC2923602

[42] Hayashida A, Inaba N, Oshima K, Nishikawa M, Shoda A, et al (2007) Re-evaluation of the true rate of hepatitis C virus mother-to-child transmission and its novel risk factors based on our two prospective studies. J Obstet Gynaecol Res 33: 417–422. 17688606 10.1111/j.1447-0756.2007.00582.x

[43] Indolfi G, Azzari C, Moriondo M, Lippi F, de Martino M, et al (2006) Alanine transaminase levels in the year before pregnancy predict the risk of hepatitis C virus vertical transmission. J Med Virol 78: 911–914. 16721858 10.1002/jmv.20640

