# Supplementary figures and images for: Variation of Transaminases, HCV-RNA Levels and Th1/Th2 Cytokine Production during the Post-Partum Period in Pregnant Women with Chronic Hepatitis C
Source: PLoS One. 2013 Oct 10;8(10):e75613. doi: 10.1371/journal.pone.0075613 (PMC3794969; doi:10.1371/journal.pone.0075613)

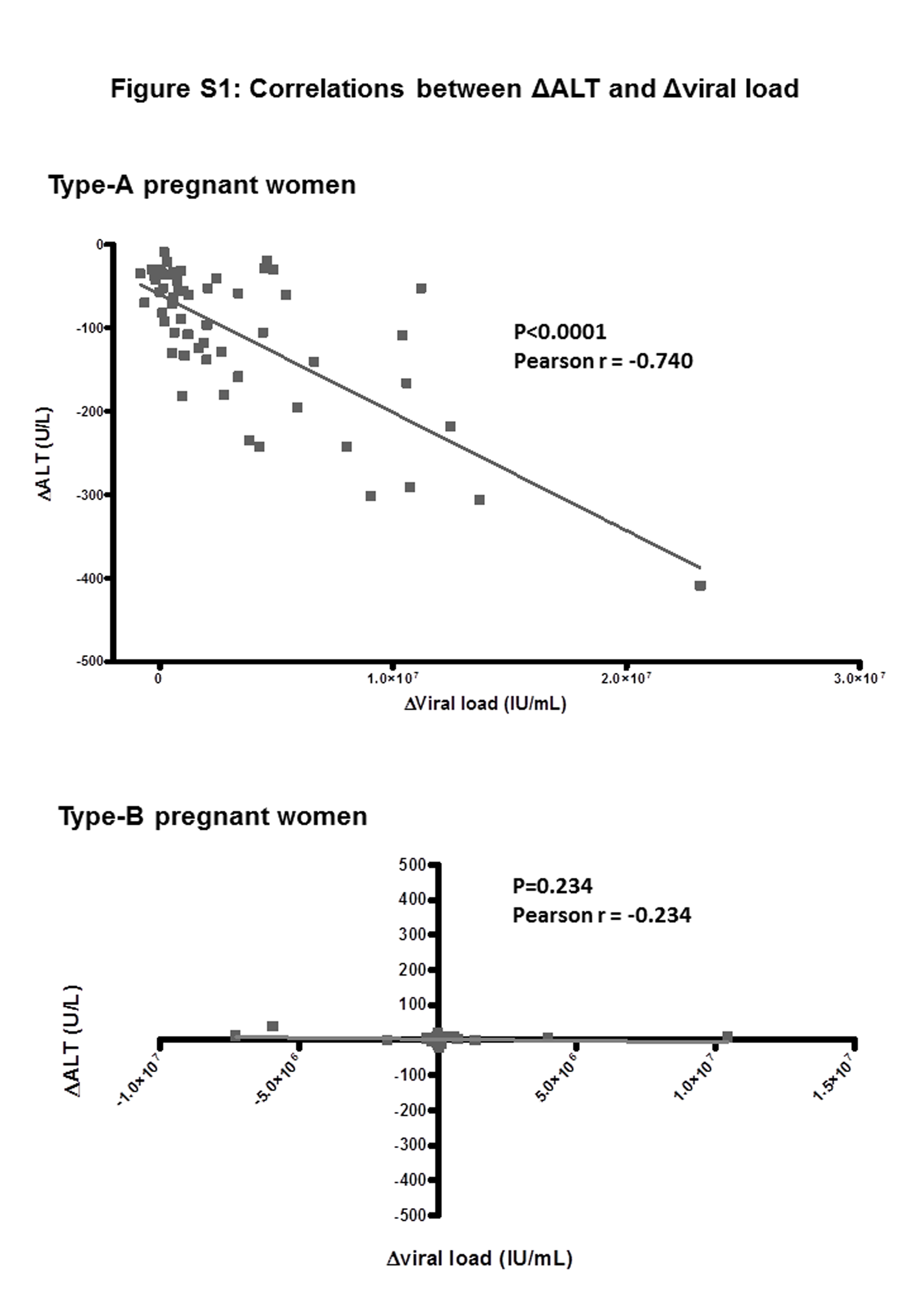

Supplement: Figure S1 — Measure of the association between ΔALT and ΔViral load. The HCV-RNA+ve mothers exhibited a significant correlation between ALT levels and viral load, while the Type-B women did not present this association. ΔViralLoad = VL3–6 months post-partum–VLpartum, ΔALT: ALT3-6 months post-partum–ALTpartum. Statistical analysis was performed using Pearson's r to measure the correlation (linear dependence) between the two variables. (TIF) [file pone.0075613.s001.tif]

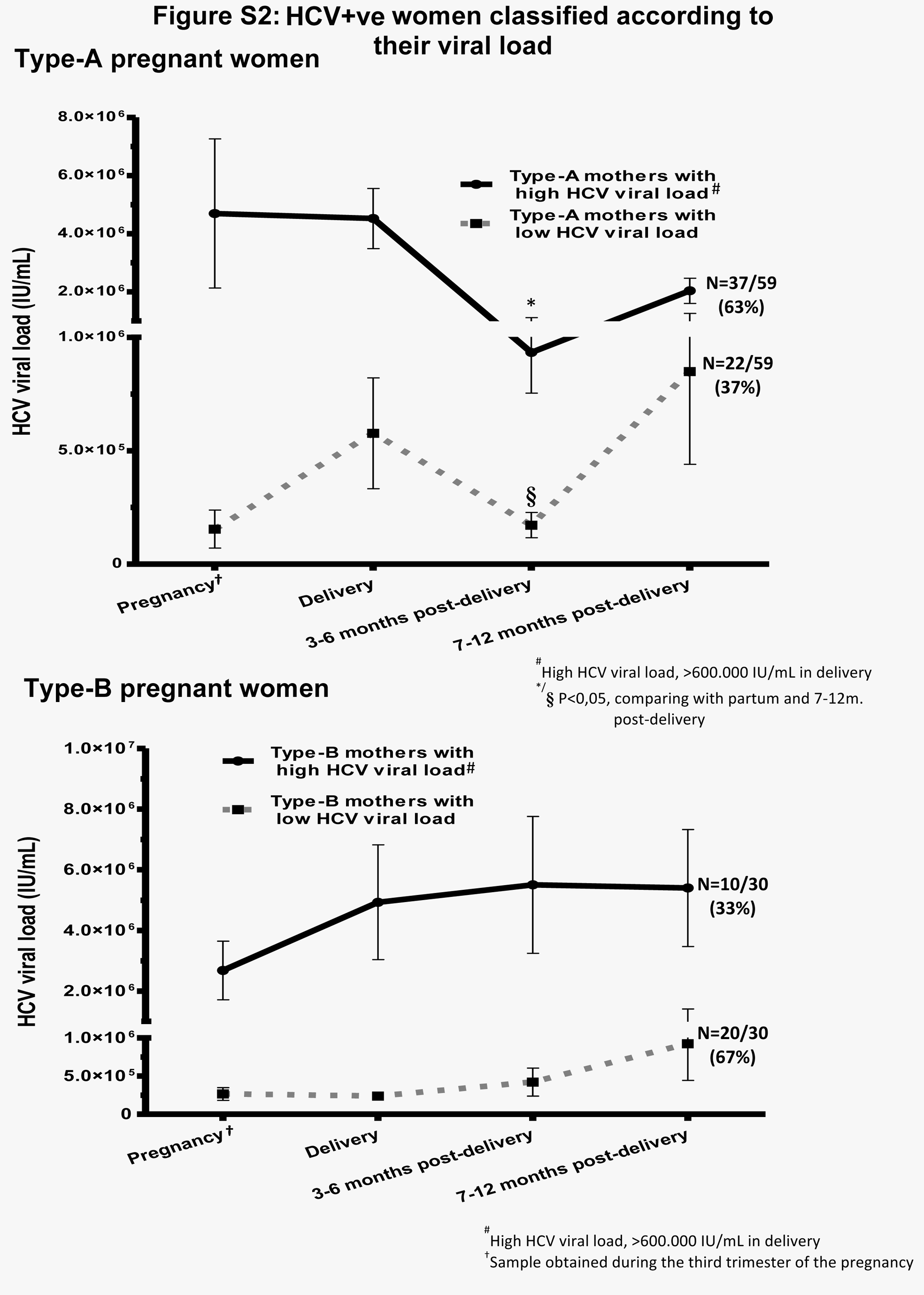

Supplement: Figure S2 — HCV-RNA+ve women classified according to their viral load. Mothers with high viral load: >600,000 IU/mL; mothers with low viral load: ≤600,000 IU/mL. Statistical analysis was performed using the paired/unpaired Student's t test for normally distributed quantitative variables and the Mann-Whitney Test for quantitative variables with a non-normal distribution. The Kolmogorov-Smirnov test was used to analyse the distribution of quantitative variables. (TIF) [file pone.0075613.s002.tif]

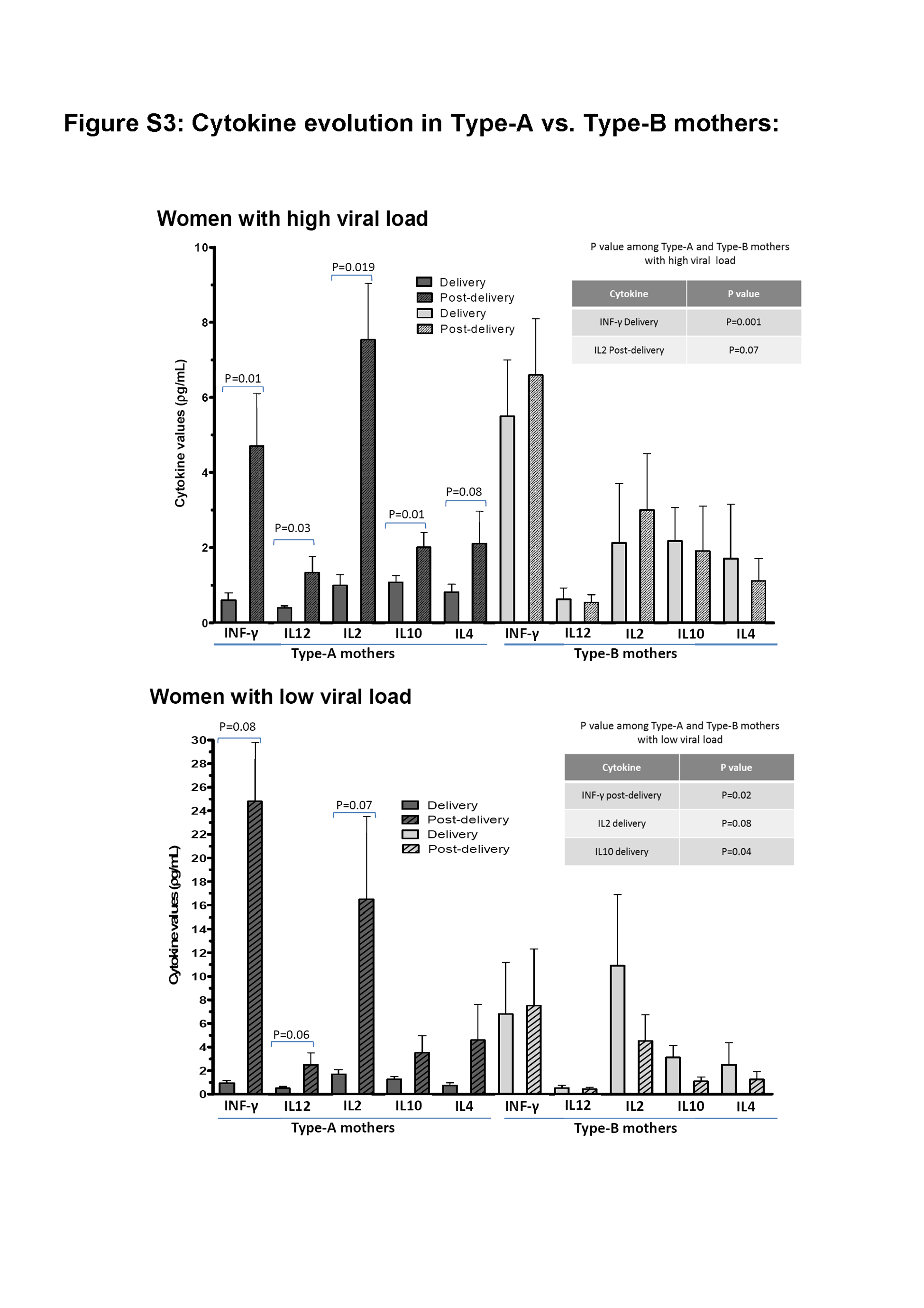

Supplement: Figure S3 — The evolution of the cytokine serum levels in HCV-RNA+ve pregnant women (Type-A vs. Type-B) categorized into high viral load and low viral load according to the serum HCV-RNA levels in the intra-partum period. Mothers with high viral load: >600,000 IU/mL; mothers with low viral load: ≤600,000 IU/mL. Statistical analysis was performed using the paired/unpaired Student's t test for normally distributed quantitative variables and the Mann-Whitney Test for quantitative variables with a non-normal distribution. (TIF) [file pone.0075613.s003.tif]

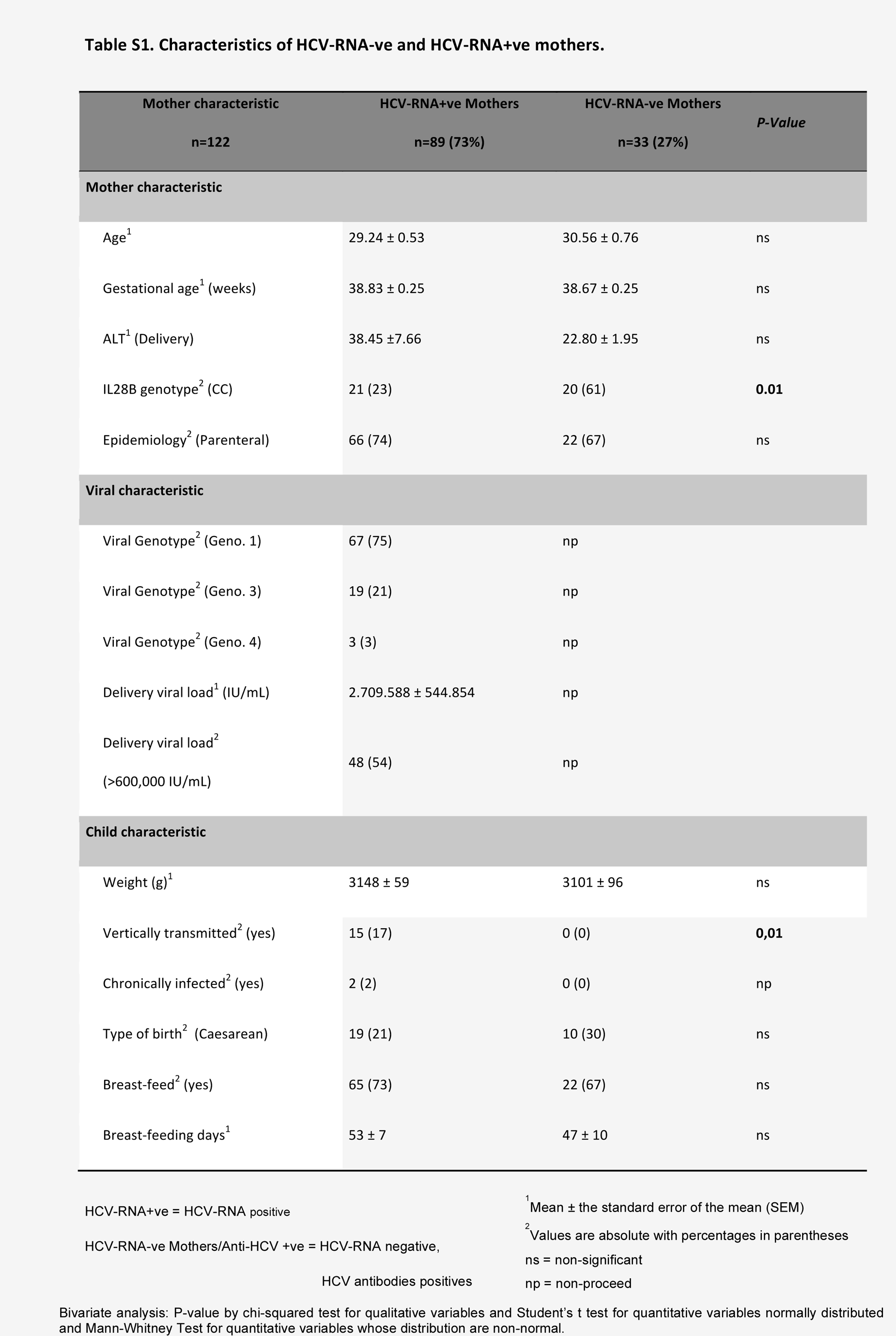

Supplement: Table S1 — Characteristics of HCV-RNA-ve and HCV-RNA+ve mothers. (TIF) [file pone.0075613.s004.tif]

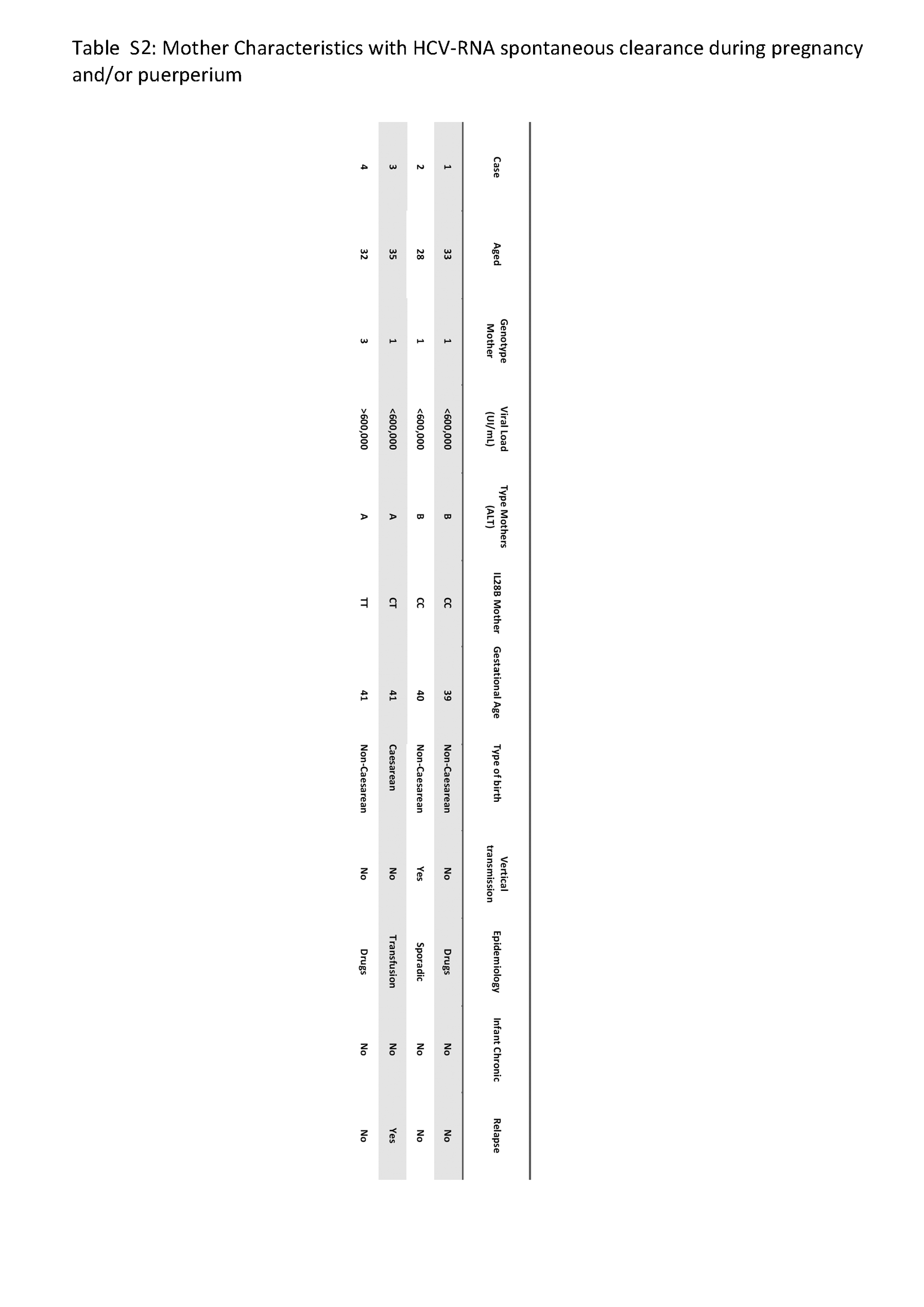

Supplement: Table S2 — Characteristics of the mothers with HCV-RNA spontaneous clearance during pregnancy and/or puerperium. (TIF) [file pone.0075613.s005.tif]
